# Tamoxifen enhances the cytotoxic effects of nelfinavir in breast cancer cells

**DOI:** 10.1186/bcr2602

**Published:** 2010-07-01

**Authors:** Ansgar Brüning, Klaus Friese, Alexander Burges, Ioannis Mylonas

**Affiliations:** 1 Department of Obstetrics and Gynaecology, Campus Innenstadt, Ludwig-Maximilians-University Munich, 11 Maistrasse, Munich 80337, Germany; 2 Department of Obstetrics and Gynaecology, Campus Grosshadern, Ludwig-Maximilians-University Munich, 15 Marchioninistrasse, Munich 81377, Germany

## Abstract

**Introduction:**

The HIV protease inhibitor nelfinavir is currently under investigation as a new anti-cancer drug. Several studies have shown that nelfinavir induces cell cycle arrest, endoplasmic reticulum stress, autophagy, and apoptosis in cancer cells. In the present article, the effect of nelfinavir on human breast cancer cells is examined and potential combination treatments are investigated.

**Methods:**

The effects of nelfinavir and tamoxifen on the human breast cancer cell lines MCF7, T47 D, MDA-MB-453, and MDA-MB-435 were tested by analysing their influence on cell viability (via 3-(4,5-dimethylthiazol-2-yl)-2,5-diphenyltetrazolium bromide assay), apoptosis (annexin binding, poly(ADP-ribose) polymerase cleavage), autophagy (autophagy marker light chain 3B expression), endoplasmic reticulum stress (binding protein and activating transcription factor 3 expression), and the occurrence of oxidative stress (intracellular glutathione level).

**Results:**

Nelfinavir induced apoptosis in all four breast cancer cell lines tested, although the extent of autophagy and endoplasmic reticulum stress varied among the cell lines. The concentration of nelfinavir needed for an efficient induction of apoptosis in breast cancer cells could be reduced from 15 μg/ml to 6 μg/ml when combined with tamoxifen. At a concentration of 6 μg/ml, tamoxifen substantially enhanced the endoplasmic reticulum stress reaction in those cell lines that responded to nelfinavir with binding protein (BiP) upregulation (MCF7, T47D), and enhanced autophagy in cell lines that responded to nelfinavir treatment with autophagy marker light chain 3B upregulation (MDA-MB-453). Although tamoxifen has been described to be able to induce oxidative stress at concentrations similar to those applied in this study (6 μg/ml), we observed that nelfinavir but not tamoxifen reduced the intracellular glutathione level of breast cancer cells within hours of application by up to 32%, suggesting the induction of oxidative stress was an early event and an additional cause of the apoptosis induced by nelfinavir.

**Conclusions:**

The results demonstrate that nelfinavir may be an effective drug against breast cancer and could be combined with tamoxifen to enhance its efficacy against breast cancer cells. Moreover, the cytotoxic effect of a tamoxifen and nelfinavir combination was independent of the oestrogen receptor status of the analysed breast cancer cells, suggesting a potential benefit of a combination of these two drugs even in patients with no hormone-responsive tumours. We therefore recommend that clinical studies on nelfinavir with breast cancer patients should include this drug combination to analyse the therapeutic efficacy as well as the safety and tolerability of this potential treatment option.

## Introduction

Breast cancer is the most frequent cancer in the female population [1]. Although tremendous progress in the treatment of breast cancer has been achieved during past decades, it is still the principal cause of cancer death for females worldwide [1,2].

Tamoxifen is a selective oestrogen receptor antagonist, and since its introduction in cancer therapy has become the standard treatment option for hormone-responsive breast cancer patients [2-5]. Not all breast cancer patients, however, benefit from an endocrine therapy with tamoxifen [3]. Interestingly, several hormone receptor-independent effects of tamoxifen have been described, leading to apoptosis when higher concentrations of tamoxifen are applied [6,7]. A combination therapy of tamoxifen with other drugs that cause synergistic anti-tumour effects might therefore be an interesting option in the therapy of breast carcinomas.

Nelfinavir (Viracept^®^; Pfizer, Groton, CT, USA) is an HIV protease inhibitor that has long been an essential component of the antiviral combination highly active antiretroviral therapy. Several recent *in vitro *studies have indicated that nelfinavir has potential anti-tumoral effects [8,9], and clinical studies on nelfinavir are ongoing to confirm its efficacy against human cancers *in vivo *[10-13]. Nelfinavir exerts pleiotropic effects on cancer cells, including induction of apoptosis, necrosis, and autophagy [9,14,15]. Nelfinavir is believed to either cross-react with a protease of the cytoplasmic proteasomal protein degradation machinery or with endoplasmic reticulum-resident proteases [15,16]. In both cases, this protease inhibition can lead to the accumulation of misfolded proteins that cause the unfolded protein response or endoplasmic reticulum stress response [17-19]. These pathways are primarily associated with a transient cell cycle arrest and upregulation of molecular chaperones such as binding protein (BiP) and other members of the heat shock family, in order to repair and prevent further cell damage [18]. A prolonged or irreparable stress reaction, however, eventually switches from a repair and survival mechanism to cell death executed by apoptosis [20,21]. This nonclassical cell death mechanism has recently become of interest because of its ability to act even on otherwise chemoresistant human cancer cells [15,22].

Since the orally available drugs tamoxifen and nelfinavir have anti-tumoral properties, a combination of these medications might be an intriguing option in the therapy of breast cancer patients. However, no data regarding their potential synergistic effects are yet available. We therefore tested the effect of nelfinavir and tamoxifen with regard to the influence on apoptosis, endoplasmic reticulum stress, autophagy, and oxidative stress in breast cancer cells with different oestrogen receptor status.

## Materials and methods

### Cells and cell culture

The breast cancer cell lines T47 D (ATCC HTB 133; oestrogen receptor-positive), MCF7 (ATCC HTB 22; oestrogen receptor-positive), MDA-MB-453 (ATCC HTB 131; oestrogen receptor-negative), and MDA-MB-435 S (ATCC HTB 129; oestrogen receptor-negative) - all kindly provided by G Saretzki (Newcastle, UK) - were cultured in RPMI-1640 medium supplemented with 10% foetal calf serum and antibiotics at 37°C in a humidified atmosphere with 5% CO_2_. All cell culture reagents were from PAA (Pasching, Austria).

### Drugs and drug treatment

Nelfinavir (Viracept^®^) was generously provided by Pfizer. Nelfinavir was dissolved in ethanol and kept at -20°C as a 100 mg/ml stock solution. Tamoxifen (Sigma, Munich, Germany) was dissolved in dimethylsulfoxide at a concentration of 100 mg/ml. In control experiments, equal amounts of dimethylsulfoxide or ethanol were added.

### Cell proliferation analysis

A total of 2 × 10^4 ^cells per well were seeded in quadruplicate in 24-well cell culture plates and were incubated with nelfinavir for up to 4 days. The number of viable, trypan blue-excluding cells was determined by a haemocytometer.

### MTT assay

For the 3-(4,5-dimethyl-2-thiazolyl)-2,5-diphenyl-2H-tetrazolium bromide (MTT) assay analysis, 20 μl MTT (Sigma) stock solution (5 mg/ml PBS) was added to viable cells in 200 μl cell culture medium for 1 hour under cell culture conditions. The water-insoluble precipitate was dissolved in 100 μl dimethylsulfoxide and analysed by an ELISA reader at 595 nm.

### Annexin binding assay

FITC-labelled annexin V (Biocat, Heidelberg, Germany) was applied to viable cells as recommended by the supplier in combination with propidium iodide, and was analysed by FACScan with an FL-1 setting (propidium iodide) at 575 nm and an FL-2 setting (FITC) at 530 nm. FACScan analysis was performed using a Becton Dickinson FACScan analyser (Becton Dickinson, Heidelberg, Germany).

### Western blot analysis

Cell extracts of cancer cells cultured in cell culture plates were prepared with radio-immunoprecipitation buffer (50 mM Tris, pH 8.0, 150 mM NaCl, 1% NP40, 0.5% doxycholine, 0.1% SDS) and 20 μg protein (BioRad Bradford Assay; BioRad, Munich, Germany) were subjected to SDS-PAGE. Proteins were transferred to polyvinylidene fluoride membranes in a BioRad Mini Protean II Cell (BioRad) at 1 mA/cm^2 ^membrane in 10% methanol, 192 mM glycine, 25 mM Tris, pH 8.2. Membranes were blocked with 4% nonfat milk powder in PBS-0.05% Tween for 4 hours. Primary antibodies were applied in blocking buffer and incubated at room temperature overnight.

Antibodies against poly(ADP-ribose) polymerase, phospho-ERK1/2 (pp44/pp42), AKT, phospho-AKT, mcl-1, IκB, and autophagy marker light chain 3B were all purchased from Cell Signaling Technology (NEB, Frankfurt, Germany). Antibodies against BiP (H-129), activating transcription factor 3 (C-19) and β-actin (C4) were from SantaCruz Biotech (Heidelberg, Germany). Secondary, alkaline phosphatase-coupled antibodies against the corresponding primary antibodies were from Dianova (Hamburg, Germany). Alkaline phosphatase detection was performed either by the chromogenic BCIP/NBT assay (Promega, Mannheim, Germany) or by the chemiluminescent alkaline phosphatase detection assay (Millipore, Schwalbach, Germany), and the results were analysed and documented using a BioRad QuantityOne Image Analyzer and documentation software (BioRad).

### Determination of intracellular glutathione levels

To detect intracellular glutathione levels, cells were seeded in 96-well cell culture dishes and allowed to grow for 24 hours under cell culture conditions. Cells were then incubated with the cytotoxic drugs for up to 5 hours. Intracellular glutathione levels were quantified using the bioluminescent Promega GSH-Glo™ glutathione assay (Promega), essentially as recommended by the supplier. In brief, adherent cells were directly dissolved in 100 μl GSH-Glo™ lysis and reaction buffer. After addition of 100 μl GSH-Glo™ Luciferin detection reagent, luminescence was detected using a MicroLumat LB 96P bioluminometer (EG&G Berthold, Bad Wildbad, Germany).

### Determination of proteasomal activity

For the determination of cellular proteasome activity, cells were seeded in 96-well cell culture dishes, allowed to grow for 24 hours under cell culture conditions, and then incubated with cytotoxic drugs for up to 5 hours. Proteasomal activity was analysed using the bioluminescent Promega Proteasome-Glo™ assay (Promega) as recommended by the supplier. Adherent cells were directly dissolved in 50 μl Proteasome-Glo™ lysis and reagent buffer, containing Suc-LLVY-aminoluciferin as a substrate. Leukaemia cells were collected by centrifugation before lysis. Bioluminescence was detected using a MicroLumat LB 96P bioluminometer (EG&G Berthold).

### Statistical analysis

All experiments, except western blots and FACScan analysis, were performed in quadruplicate. The results were evaluated using the nonparametric Wilcoxon sum test and the Mann-Whitney U rank-sum test where applicable (PASW version 17.0; SPSS Inc., Chigaco, IL, USA). Values were plotted as the mean ± standard deviation, and significance was assumed at *P *< 0.05 using the two-tailed test. Significant relations are indicated in the figures and the statistical test used is mentioned in the corresponding figure legend.

### Ethical aspects

All experiments were performed on established cancer cell lines. No ethical approval or informed consent was thus needed.

## Results

### Nelfinavir reduces cell proliferation of breast cancer cells and is able to induce apoptosis in breast cancer cells when applied at higher concentrations

**Figure 1:**
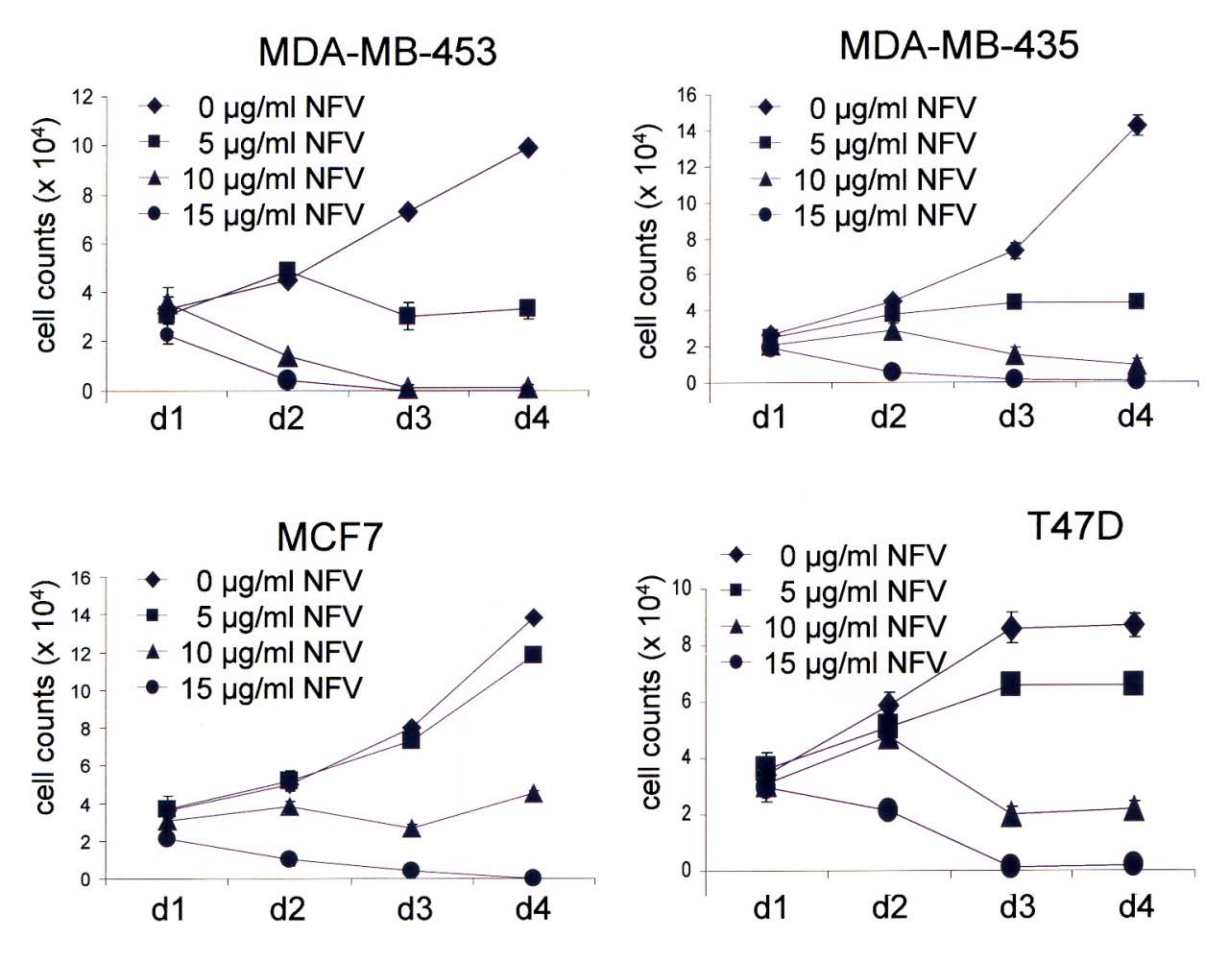
**Effect of nelfinavir on the cell survival of breast cancer cells**. A total of 2 × 10^4 ^cells per well of the four indicated breast cancer cell lines were seeded in quadruplicate in 24-well cell culture plates, incubated with the indicated nelfinavir (NFV) concentrations, and the number of trypan blue-excluding cells was determined over a period of 4 days (d1 to d4). Values represent the mean ± standard deviation.

The human breast cancer cell lines T47 D, MCF7, MDA-MB-453, and MDA-MB-435 were incubated with nelfinavir at various concentrations with various time intervals and were analysed for cell proliferation by counting viable cells. Low doses of nelfinavir (5 μg/ml) reduced the cell proliferation of breast cancer cells, and complete cell death was achieved at a concentration of 15 μg/ml (Figure 1). Nelfinavir acted on oestrogen receptor-positive (T47 D, MCF7) as well as on oestrogen receptor-negative (MDA-MB-453, MDA-MB-435) breast cancer cell lines.

### Tamoxifen enhances the cytotoxic effect of nelfinavir

The mean plasma concentration of nelfinavir in HIV-infected persons taking oral doses of nelfinavir was determined to be 2.2 μg/ml, reaching maximal plasma concentrations of up to 4 μg/ml [23,24]. These concentrations can achieve only a partial reduction of breast cancer cell proliferation (Figure 1), and are not efficient in inducing apoptosis in breast cancer cells. The plasma concentration of nelfinavir, however, can be significantly increased by administering higher oral doses of nelfinavir or by intravenous application of nelfinavir [25]. Still, a possible combination strategy of nelfinavir with other chemotherapeutic drugs that would allow a reduction of single drug concentrations would be advantageous.

**Figure 2:**
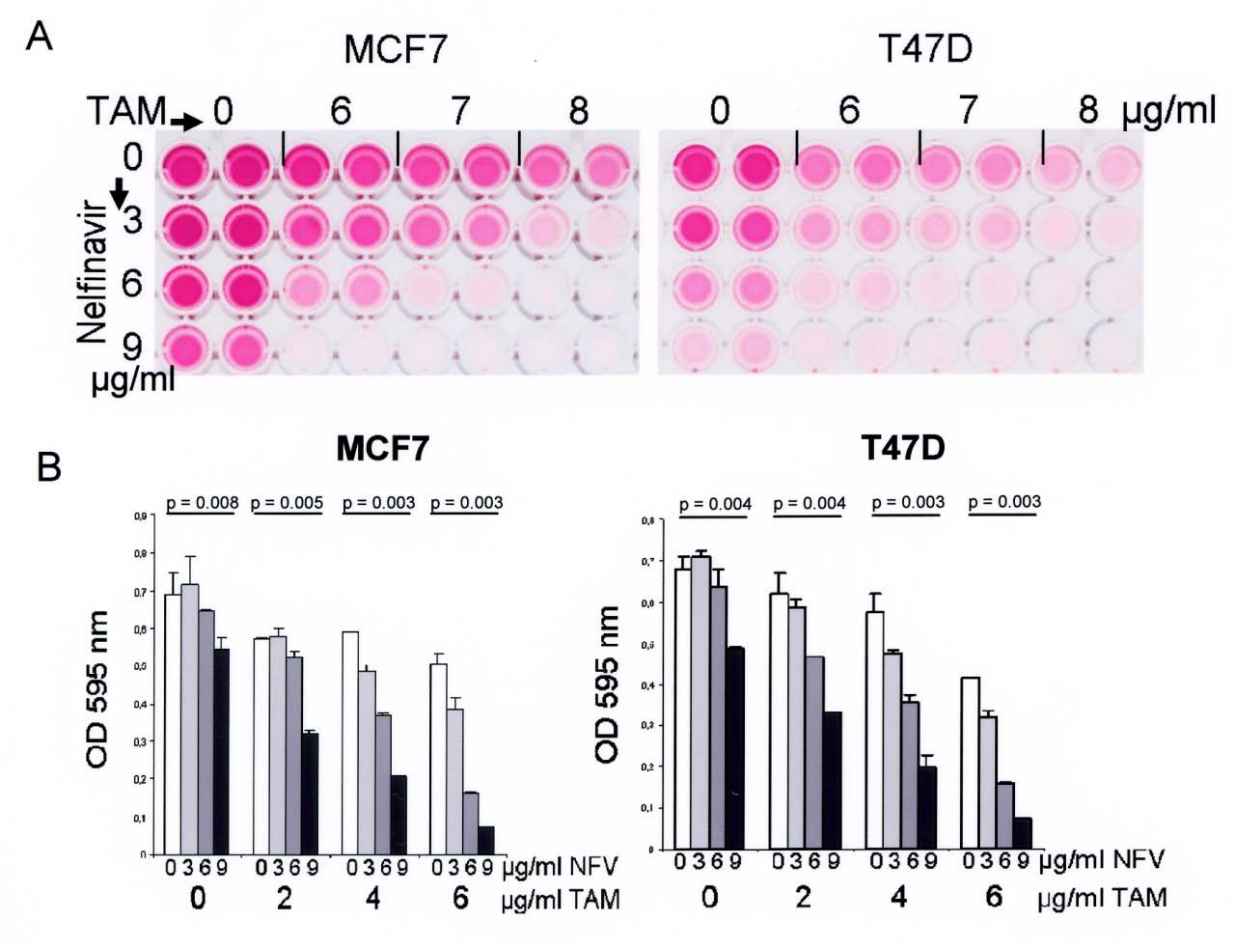
**Combination effect of nelfinavir and tamoxifen on the cell survival of breast cancer cells**. **(a) **A total of 5 × 10^3 ^MCF7 and T47 D cells per well were seeded in quadruplicate in 96-well cell culture plates, incubated with the indicated nelfinavir and tamoxifen (TAM) concentrations either alone or in combination, and were analysed for cell viability by an MTT assay after 72 hours of incubation. **(b) **A similar experiment was performed using different nelfinavir and TAM concentrations, and MTT-derived staining intensities were analysed by a photometer (shown as bar graphs). Values represent the mean ± standard deviation. Significance assumed at *P *< 0.05 with the nonparametric Wilcoxon rank-sum test.

**Figure 3:**
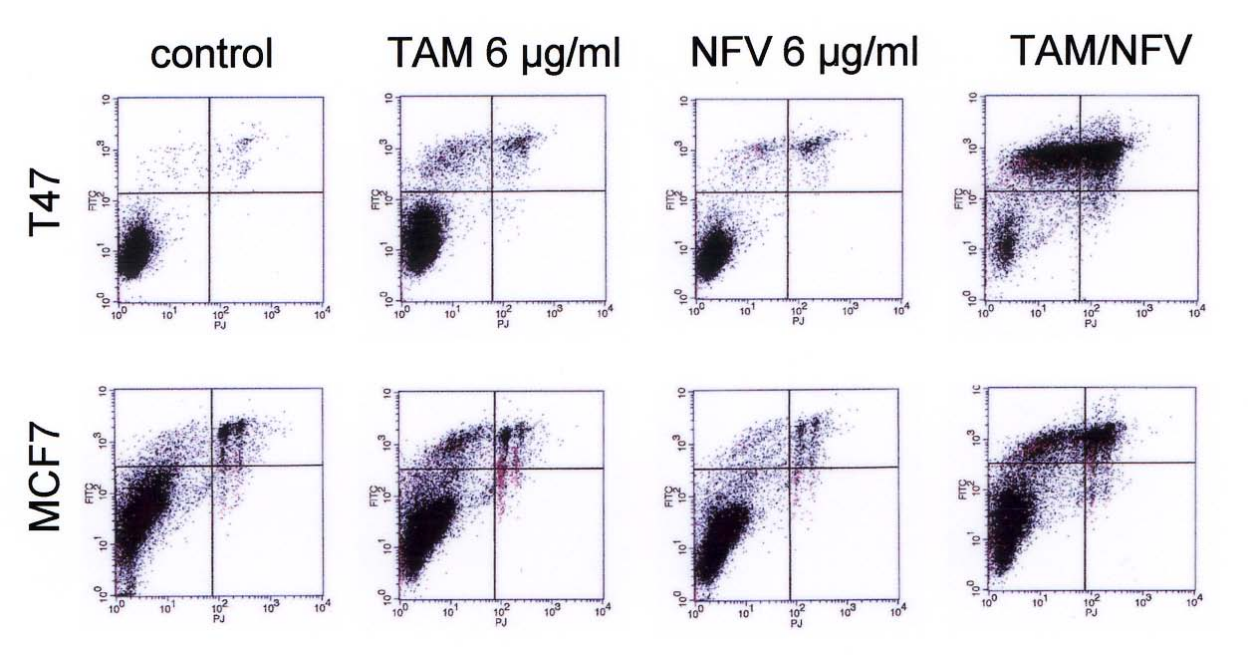
**Combination of nelfinavir and tamoxifen enhances apoptosis in breast cancer cells**. The breast cancer cell lines MCF7 and T47 D were incubated with the indicated nelfinavir (NFV) and tamoxifen (TAM) concentrations either alone at 6 μg/ml or in combination (6 μg/ml NFV plus 6 μg/ml TAM), and after 48 hours of incubation were analysed by FACScan analysis for the occurrence of apoptosis (FITC-annexin binding) and necrosis (propidium iodide permeability). FL-1 setting (propidium iodide; PJ), 575 nm; FL-2 setting (FITC), 530 nm.

When combining nelfinavir with tamoxifen, we observed a substantially enhanced induction of cell death even at lower nelfinavir concentrations (Figure 2). For example, when used as a single agent, 6 μg/ml nelfinavir induced only a slight and nonsignificant reduction of cell viability by 6.1% in MCF7 cells and by 6.4% in T47 D cells (Figure 2). Tamoxifen at 6 μg/ml reduced the cell viability by 26.5% in MCF7 cells and by 40% in T47 D cells (Figure 2; *P *< 0.05). The combination of both nelfinavir and tamoxifen, however, significantly reduced cell viability of MCF7 cells and T47 D cells by up to 77.0% and 76.8%, respectively (Figure 2; *P *< 0.05). FACScan analysis confirmed that the combination of 6 μg/ml nelfinavir with 6 μg/ml tamoxifen efficiently induced apoptosis in breast cancer cells (Figure 3 and Table 1), although the same concentrations proved to be insufficient for the induction of apoptosis when used as single agents (Figure 3 and Table 1).

**Table 1:** Quantitative analysis of apoptotic and necrotic cells after tamoxifen and nelfinavir treatment

Cell line	LL (%)	UL (%)	UR (%)	LR (%)
T47D				
Control	98.78	0.44	0.77	0.03
TAM 6 μg/ml	92.71	4.42	2.71	0.17
NFV 6 μg/ml	95.50	2.17	2.31	0.03
TAM/NFV	11.18	43.05	44.44	1.34
MCF7				
Control	88.04	3.10	7.52	1.33
TAM 6 μg/ml	83.05	7.16	6.89	2.90
NFV 6 μg/ml	92.80	2.46	3.80	0.95
TAM/NFV	63.68	14.63	19.48	2.21

The relative cell distributions among the quadrants shown in Figure 2 were analysed using the BectonDickinson CellQuest software. LL, lower left (unstained; viable); UL, upper left (annexin-positive; apoptotic); UR, upper right (annexin-positive and propidium iodide-positive; apoptotic/necrotic); LR, lower right (propidium iodide permeable; necrotic); TAM, tamoxifen; NFV, nelfinavir.

### Nelfinavir exerts pleiotropic proapoptotic and anti-apoptotic effects in breast cancer cells

**Figure 4:**
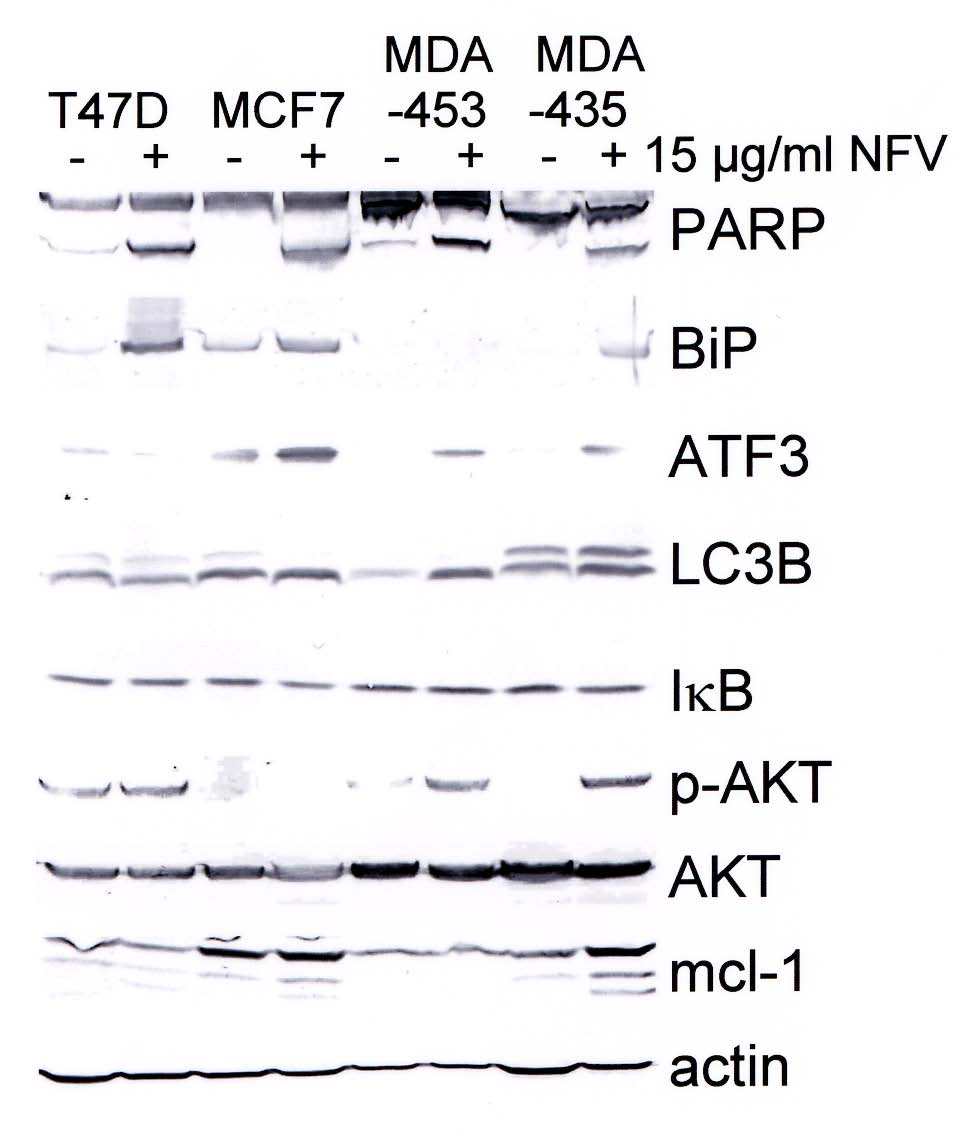
**Treatment of breast cancer cells with nelfinavir activates pleiotropic pathways**. The indicated four breast cancer cell lines were incubated with or without 15 μg/ml nelfinavir (NFV) for 48 hours and were analysed by Western blots for the expression and modification of cell survival-related proteins and pathways. PARP, poly(ADP-ribose) polymerase; BiP, binding protein; ATF3, activating transcription factor 3; LC3B, autophagy marker light chain 3B.

To gain a better insight into the cell death mechanism induced by nelfinavir alone and in combination with tamoxifen, Western blot analyses of drug-treated breast cancer cells were performed. First, the effects of nelfinavir as a single agent on breast cancer cells were analysed. Using the cleavage of poly(ADP-ribose) polymerase as an indicator of apoptosis, the specific induction of apoptosis in breast cancer cells by high nelfinavir concentrations (15 μg/ml) could be confirmed (Figure 4). Western blotting further showed upregulation of BiP and activating transcription factor 3 (ATF3) in nelfinavir-treated breast cancer cells, indicating induction of endoplasmic reticulum stress. Upregulation of BiP following nelfinavir treatment, however, was scarcely detectable in MDA-MB-453 cells. In these cells, strong upregulation of autophagy marker light chain 3B could be observed instead.

In all four cell lines, no change in the expression of the proteasome-regulated NF-κB inhibitor IκB could be observed, suggesting no influence of nelfinavir on the proteasome activity in breast cancer cells. We have previously described an upregulation of the anti-apoptotic mcl-1 protein by nelfinavir in ovarian cancer cells [26], but this could only be observed in one (MDA-MB-435) of the four tested breast cancer cell lines. Nelfinavir has further been described as reducing AKT phosphorylation, resulting in enhanced radiosensitivity [11,16] - which could be of special importance for breast cancer treatment, for which radiotherapy can be applied. We observed no inhibition of AKT signalling in MCF7 and T47 D cells by nelfinavir, however, and we even observed a markedly enhanced AKT phosphorylation level in nelfinavir-treated MDA-MB-453 and MDA-MB-435 cells (Figure 4).

### Tamoxifen enhances the pleiotropic effects of nelfinavir in breast cancer cells

**Figure 5:**
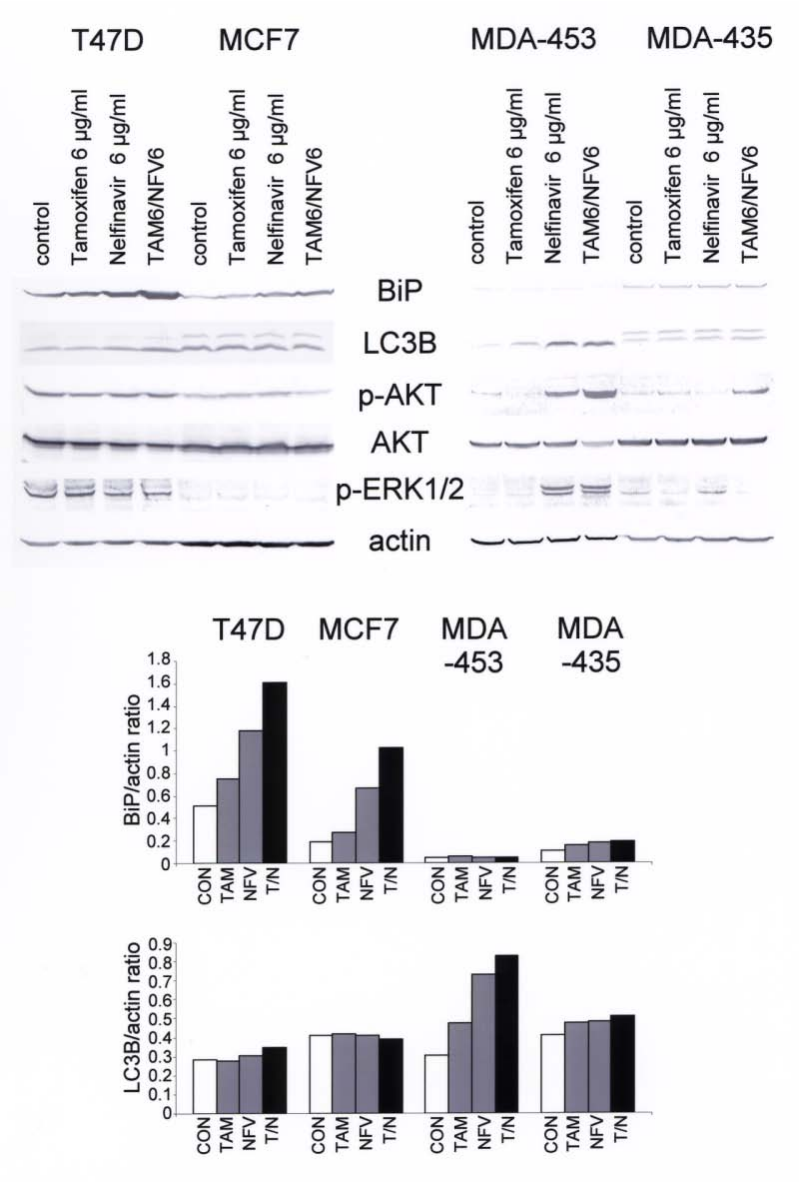
**Combination of nelfinavir and tamoxifen enhances the effect of nelfinavir-induced pathways**. The oestrogen receptor-positive breast cancer cell lines MCF7 and T47 D and the oestrogen receptor-negative breast cancer cell lines MDA-MB-435 and MDA-MB-453 were incubated with the indicated nelfinavir (NFV) and tamoxifen (TAM) concentrations for 48 hours and were analysed by Western blotting. Staining intensities of selected bands were analysed using a gel documentation system and program (BioRad Quantity One; BioRad, Munich, Germany), and were related to the corresponding β-actin expression value as an internal control (CON). For autophagy marker light chain 3B (LC3B) expression, the lower LC3B-II band was used for calculations. BiP, binding protein; T/N, tamoxifen and nelfinavir combination.

The effects of tamoxifen on the proapoptotic and anti-apoptotic pathways that were induced by nelfinavir (Figure 4) were further investigated. Cell lysates of breast cancer cells treated with 6 μg/ml nelfinavir or 6 μg/ml tamoxifen alone or in combination were subjected to western blot analysis (Figure 5). Tamoxifen enhanced upregulation of BiP in T47 D and MCF7 cells, and to a lesser in MDA-MB-435 and MDA-MB-453 cells (Figure 5). In MDA-MB-453 cells, expression of the autophagy marker light chain 3B was strongly enhanced. Tamoxifen, nelfinavir, and its combination had no effect on AKT or ERK phosphorylation in T47 D and MCF7 cells but, in MDA-MB-435 and MDA-MB-453 cells, the combination of tamoxifen with nelfinavir markedly enhanced AKT phosphorylation (Figure 5).

### Nelfinavir reduces glutathione levels in breast cancer cells

**Figure 6:**
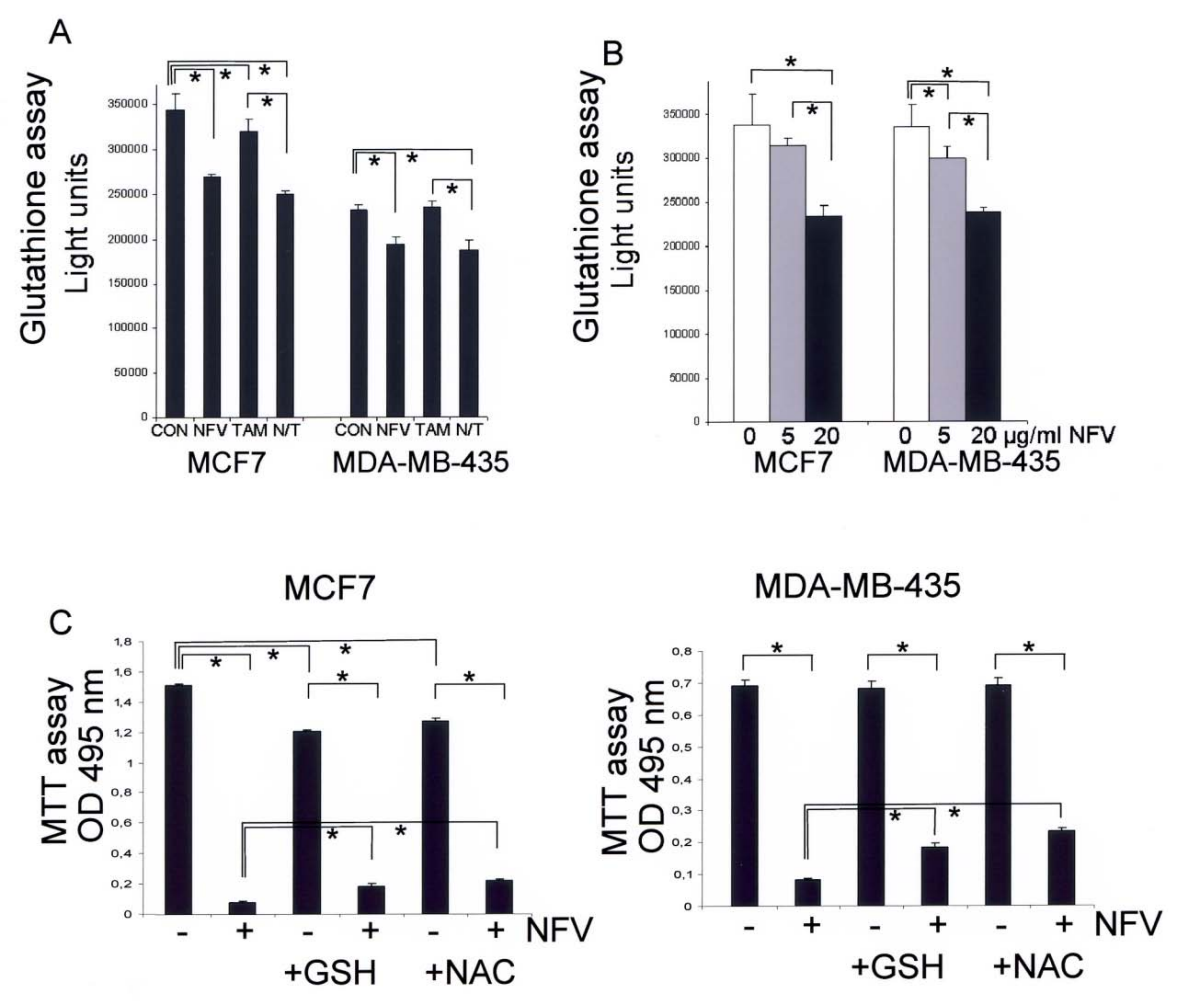
**Nelfinavir, but not tamoxifen, induces glutathione reduction in breast cancer cells. (a) **A total of 5 × 10^3 ^MCF7 and MDA-MB-435 cells per well were seeded in 96-well cell culture dishes and were incubated for 5 hours with either 6 μg/ml nelfinavir (NFV) or 6 μg/ml tamoxifen (TAM) alone or in combination (N/T). Intracellular glutathione levels were quantified using the bioluminescent Promega GSH-Glo™ glutathione assay (Promega, Mannheim, Germany). **(b) **MCF7 and MDA-MB-435 cells were incubated for 5 hours with the indicated NFV concentrations and were analysed for the cellular glutathione level as described in (a). **(c) **MCF7 and MDA-MB-435 cells were incubated with or without 15 μg/ml NFV for 72 hours in the presence or absence of 5 mM glutathione (GSH) or 5 mM *N*-acetyl-cysteine (NAC), and were analysed for cell survival by an MTT assay. Values represent the mean ± standard deviation. *Significance assumed at *P *< 0.05 with the nonparametric Mann-Whitney U rank-sum test. CON, control.

Tamoxifen has been reported to induce oxidative stress [6]. Oxidative stress is reflected by or can be facilitated by reduced intracellular glutathione levels, because glutathione serves as an endogenous antioxidant and reduced glutathione levels facilitate apoptosis [27]. When breast cancer cells were incubated with tamoxifen or nelfinavir, we observed that nelfinavir reduced glutathione levels in breast cancer cells even more than tamoxifen did (Figure 6a). At a concentration of 6 μg/ml, nelfinavir reduced the intracellular glutathione level of MCF7 and MDA-MB-435 cells by 21.6% and 16.6%, respectively (*P *< 0.05). A slight but statistically significant reduction of the glutathione level by 7% (*P *< 0.05) could be observed for MCF7 cells treated with 6 μg/ml tamoxifen (Figure 6a). The combination of 6 μg/ml nelfinavir with 6 μg/ml tamoxifen, however, did not further reduce the glutathione levels in breast cancer cells in a significant manner (Figure 6a). Since the subtoxic concentration of 6 μg/ml nelfinavir was already effective in reducing intracellular glutathione levels, we tested the effect of nelfinavir at higher concentrations (20 μg/ml); these higher levels proved to be toxic to all of the four breast cancer cell lines investigated. Figure 6b shows that 20 μg/ml nelfinavir reduced intracellular glutathione levels by up to 32.4% in MCF7 cells and by 30.1% in MDA-MB-435 cells.

The intracellular glutathione levels might vary not only because of external drug applications, but likewise due to differences in cell growth, nutrient concentrations (especially that of cysteine), and the redox state within the cells or of the surrounding medium. These factors can vary under cell culture conditions. Exogenously applied glutathione or cysteine derivatives such as *N*-acetyl-cysteine can enhance or replenish intracellular glutathione levels or support the antioxidative effect of glutathione. To analyse the involvement of glutathione depletion on nelfinavir-induced apoptosis, intracellular glutathione contents were replenished by the addition of externally applied glutathione or *N*-acetyl-cysteine. Figure 6c demonstrates that the addition of glutathione, and even more so the addition of *N*-acetyl-cysteine, can attenuate the cytotoxic effect of nelfinavir on breast cancer cells. In the absence of external glutathione or *N*-acetyl-cysteine, only 5% of the MCF7 cells and 12% of the MDA-MB-435 cells survived the application of 15 μg/ml nelfinavir (Figure 6c). Although the addition of 5 mM glutathione was not without negative effects on the cell viability of MCF7 cells (Figure 6c), the presence of external glutathione enhanced the remaining cell viability of MCF7 cells to 15% and to 17% in the case of MDA-MB-435 cells (Figure 6c). In the presence of 5 mM *N*-acetyl-cysteine, 18% of the MCF7 cells remained viable, and up to 34% of the MDA-MB-435 cells survived the addition of nelfinavir (Figure 6c).

### Nelfinavir has no effect on the chymotrypsin-like proteasome activity of breast cancer cells

Some previous studies have indicated the inhibition of proteasomal activity by HIV protease inhibitors, including nelfinavir [26,28]. The fact that nelfinavir has no influence on expression of the proteasome-regulated NF-κB inhibitor IκB (Figure 4), however, indirectly suggests that nelfinavir has no effect on the proteasomal activity of breast cancer cells.

**Figure 7:**
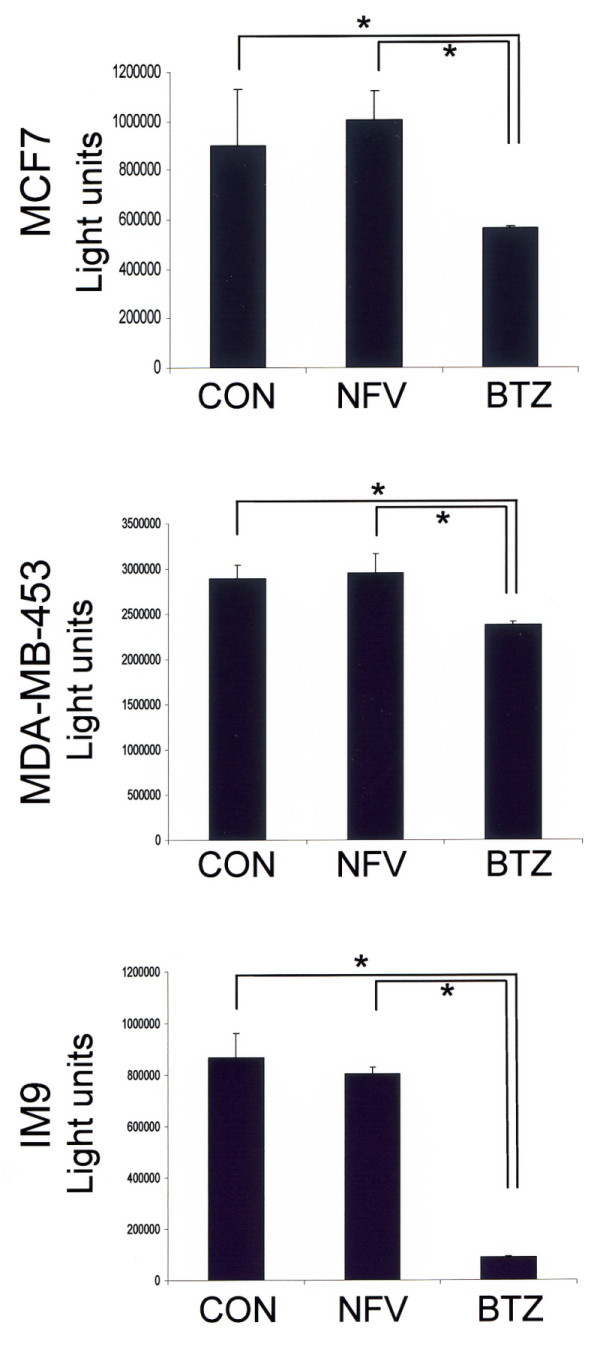
**Nelfinavir has no effect on the chymotrypsin-like proteasome activity of breast cancer cells**. A total of 5 × 10^3 ^MCF7 and MDA-MB-435 cells per well were seeded in quadruplicate in 96-well cell culture dishes and were incubated for 5 hours with either 15 μg/ml nelfinavir (NFV) or 15 ng/ml bortezomib (BTZ). Chymotryptic proteasomal activity was analysed using the bioluminescent Promega Proteasome-Glo™ assay (Promega, Mannheim, Germany) with Suc-LLVY-aminoluciferin as a substrate. The BTZ-sensitive myeloic leukaemia cell line IM9 was used as a BTZ-responsive control and was treated with 8 μg/ml NFV and 4.5 ng/ml BTZ. Values represent the mean ± standard deviation. *Significance assumed at *P *< 0.05 with the nonparametric Mann-Whitney U rank-sum test. CON, control.

To clearly detect any influence of nelfinavir on proteasomal activity in breast cancer cells, a direct, bioluminescent proteasome assay was performed. Bortezomib, a specific proteasome inhibitor clinically approved for the treatment of multiple myeloma, was used as a positive control. Figure 7 shows that nelfinavir exerts no significant effect on the chymotryptic proteasome activity in MCF7 and MDA-MB-453 breast cancer cells. The effect of bortezomib on the tested breast cancer cells was likewise poor, however, and 15 ng/ml bortezomib reduced the chymotrypsin-like activity of the proteasome in MCF7 cells by 37.5% and in T47 D cells by 17.9% only (Figure 7). Under these conditions, but at even lower bortezomib concentrations (4.5 ng/ml), a marked 89.6% loss in cell viability could be observed in IM9 cells, a bortezomib-sensitive lymphoblastoid cell line. Still, even in IM9 cells, nelfinavir displayed no significant effect on the chymotrypsin-like proteasome activity (Figure 7).

## Discussion

The HIV protease inhibitor nelfinavir is a prospective new anti-cancer drug, as shown by several *in vitro *as well as *in vivo *studies [8-13]. The concentrations of nelfinavir needed to induce cell death of cancer cells are higher than those applied for HIV-infected individuals for HIV suppression, but this may be achieved by the application of higher oral or intravenous doses of nelfinavir [25]. Still, the prospects of nelfinavir as an anti-cancer drug will rely less on its efficacy as a single drug and more on its ability to cooperate with or sensitise to other chemotherapeutic drugs or cancer treatment options. For example, we have recently demonstrated that nelfinavir cooperates with the multiple kinase inhibitor sorafenib to induce apoptosis in various cancer cell types [26,29], and enhances TNF-related apoptosis inducing ligand sensitivity in ovarian cancer cells [30].

The present results show that the cytotoxic effects of nelfinavir on breast cancer cells can be enhanced by combination with tamoxifen, thus allowing the effective concentration of nelfinavir to be reduced. Tamoxifen, although originally designed and applied as a selective oestrogen receptor modulator, also represents a drug with several described pleiotropic anti-tumoral effects [6,7] - and two recent and independent studies observed that tamoxifen is able to induce the endoplasmic reticulum stress reaction [31,32], thus explaining the synergistic effect of nelfinavir and tamoxifen on the induction of endoplasmic reticulum stress. The nelfinavir-boosting effect of tamoxifen was obviously independent of its ability to induce oxidative stress [6]. Instead, we observed that nelfinavir itself reduced cellular glutathione levels, indicating the occurrence of oxidative stress after nelfinavir treatment. Induction of oxidative stress occurs within a few hours as an early effect of nelfinavir treatment and has so far been neglected as an additional mechanism of the pleiotropic anti-cancer effects of nelfinavir. The observation that the effect of nelfinavir can be attenuated by the addition of antioxidants (glutathione or *N*-acetyl-cysteine) could have an impact on the efficacy of nelfinavir in cancer cells, as well as on the nelfinavir-induced adverse effects occurring in HIV-infected persons.

Nelfinavir has been reported to exert a radiosensitising effect by inhibiting proteasome activity and AKT signalling [16]. Inhibition of proteasomal activity or AKT signalling in breast cancer cells, however, was not observed in the present study. In contrast, nelfinavir markedly enhanced AKT phosphorylation in some breast cancer cell lines (MDA-MB-453 cells and MDA-MB-435 cells). This observation is not surprising, however, since we previously demonstrated activation of the cell-protective ERK1/2 signalling pathway by nelfinavir [26]. The endoplasmic stress reaction is primarily a cell-protective mechanism, aiming to rescue cells from transient stress-induced cell damage [21,33]. Longer exposure to cell stress mechanisms or a cellular inability to cope with the stress-induced cell damage then finally induces a switch from cell protection to autophagy and apoptosis [21,33]. Endoplasmic reticulum stress has repeatedly been shown to induce activation of both ERK1/2 and AKT signalling [34-37]. Several studies, however, have likewise shown that AKT activation, which can occur directly at the endoplasmic reticulum [38], primarily represents a short-term effect, and prolonged exposure of cells to endoplasmic reticulum stress indeed induces AKT inactivation [38,39]. In fact, we observed a reduced AKT phosphorylation when breast cancer cells were treated with nelfinavir for more than 48 hours (data not shown), although this indicates downregulation of AKT phosphorylation as a secondary event. The present data therefore do not exclude the potential use of nelfinavir as a radiosensitiser even for breast cancer patients, but a potential negative interaction between these two treatment options, especially shortly after nelfinavir application, should be kept in mind.

In addition to the data on AKT signalling, the present data revealed some other differences to previous studies performed by us and other workers on different cancer cell types. For example, upregulation of the anti-apoptotic mcl-1 protein by nelfinavir - as we observed in ovarian cancer cells [26] and leukaemia cells [29] - could only be observed in a single breast cancer cell line (MDA-MB-435 cells), and only at high concentrations of nelfinavir (Figure 4). Further, we could not demonstrate proteasome inhibition by nelfinavir in breast cancer cells.

Although nelfinavir induced cell death in all four breast cancer cell lines tested, the data presented further indicate that the cell lines respond quite differently to nelfinavir, especially regarding the effect on cell stress, autophagy, and apoptosis. This variation might be due to the different hormone receptor status of the cells, but likewise may be due to the different malignancies of the tumours from which these cell lines have been derived. We therefore tried to include various types of breast cancer cell lines in the present study, ranging from hormone receptor-positive breast cancer cells of a high differentiation grade (T47 D cells) to highly de-differentiated hormone receptor-negative breast cancer cells (MDA-MB-435 cells). Interestingly, especially when low doses of nelfinavir were applied, the de-differentiated hormone receptor-negative breast cancer cell lines (MDA-MB-453 and MDA-MB-435 cells) appeared to react even better to nelfinavir than the T47 D and MCF7 cells (Figure 1).

We observed that the combination of tamoxifen and nelfinavir was able to induce cell death in oestrogen receptor-positive as well as in oestrogen receptor-negative breast cancer cell lines. This indicates that both oestrogen receptor-positive as well as oestrogen receptor-negative breast cancer patients could benefit from a combination of these two drugs.

Since both nelfinavir and tamoxifen have to be used at concentrations higher than those used to inhibit the HIV protease in HIV-infected persons or the oestrogen receptor in hormone receptor-positive breast cancer patients, however, care has to be taken that no unexpected adverse effects occur - especially when both drugs, although displaying moderate and tolerable adverse effects as single agents, are combined. Further, the observed reduction in glutathione levels by nelfinavir might cause an unexpected drug sensitisation in other tissues.

Clinical studies on breast cancer patients testing the described combination of nelfinavir and tamoxifen are thus of high interest in order to assess both efficacy and safety of this drug combination.

## Conclusions

The present study demonstrates the efficacy of nelfinavir in breast cancer cells as a single agent, and a possible combination treatment with tamoxifen. Both nelfinavir and tamoxifen are already-approved drugs with known pharmacokinetics, and they generally exhibit relative mild and well tolerable adverse effects even after long-term application. Since the concentrations of both drugs have to be increased for an efficient cancer therapy, and a combination of these two drugs has not yet been tested in humans, however, it is important to first test the safety and tolerability of this combination in phase I studies.

## Abbreviations

ATF3: activating transcription factor 3; BiP: binding protein; ELISA: enzyme-linked immunosorbent assay; ERK: extracellular signal-regulated kinase: FACS: fluorescence-activated cell sorting; FITC: fluorescein isothiocyanate; MTT: 3-(4,5-dimethylthiazol-2-yl)-2,5-diphenyltetrazolium bromide; NF: nuclear factor; PBS: phosphate-buffered saline; TNF: tumour necrosis factor.

## Competing interests

The authors declare that they have no competing interests.

## Authors' contributions

ABr designed and coordinated the experiments, KF and ABu helped to draft the manuscript, and IM helped to draft the manuscript and performed the statistical analysis. All authors read and approved the final manuscript.
